# Public health implications of antibiotic resistance in sewage water: an epidemiological perspective

**DOI:** 10.1186/s40643-024-00807-y

**Published:** 2024-09-28

**Authors:** Kashif Rahim, Muhammad Naveed Nawaz, Mazen Almehmadi, Meshari A. Alsuwat, Luo Liu, Changyuan Yu, Shahin Shah Khan

**Affiliations:** 1 College of Life Science and Technology, Beijing University of Chemical Technology, Beijing, 100029 China; 2 Department of Biological Sciences and Technology, China University of Geosciences, Wuhan, China; 3 Department of Clinical Laboratory Sciences, College of Applied Medical Sciences, Taif University, P.O. Box 11099, Taif, 21944 Saudi Arabia

**Keywords:** Global health, Antibiotic resistance, Sewage water

## Abstract

The emergence and rapid spread of antibiotic resistance pose a major threat to global health, attributing to misuse and overuse of antibiotics resulting in antibiotics-resistant bacteria through natural mutation or transfer of resistance genes. A cross-sectional study was carried out, in which a total of 36 samples were systematically collected; of these, 26 were derived from the wastewater efflux and 10 from the receiving waters at several critical junctures along the Sutlej River. Herein, this study elucidated elevated levels of antibiotic resistance among bacterial isolates sourced from urban wastewater. *Escherichia coli* (*E. coli*) was the highest at 90% among the isolates, followed by *Klebsiella pneumoniae* (*K. pneumoniae*) at 58%, *Pseudomonas aeruginosa* (*P. aeruginosa*) at 55%, and *Salmonella spp*. at 53%. Many antibiotics were found to be more resistant including Ciproflaxacin, Co-Trimaxazole, Ampicillin and Tetracycline. Several antibiotic-resistance genes were found in isolated bacterial spp., such as Aminoglycosides (aadA), Sulfonamides (Sul1, Sul3), Tetracyclines (Tet (A/B/D)) and Cephalosporins (Bla_CTM X) at 41%, 35%, 29% and 12% respectively. Furthermore, the development of innovative wastewater treatment models and surveillance programs are crucial to counteract the dissemination of antibiotic resistance. To investigate the genetic determinants of antibiotic resistance, molecular analysis was performed, including DNA isolation, PCR amplification, and sequence analysis. The study helps investigate a diverse range of ARBs and ARGs in wastewater, which highlights the need of better laws for antibiotic usage and wastewater treatment processes. This investigation also stresses on regular monitoring of ARBs and ARGs in sewage wastewater. Through proactive interventions and sustained scientific inquiry, we can strive toward preserving environmental integrity and public health for successive generations.

**Graphical Abstract:**

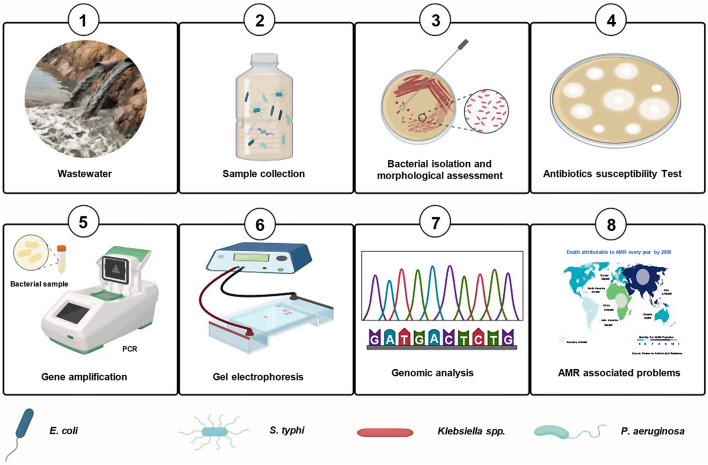

**Supplementary Information:**

The online version contains supplementary material available at 10.1186/s40643-024-00807-y.

## Introduction

Global health is constantly under the major threat of the quickly emerging and spreading antibiotic resistant bacterial strains which is due to the bacterial acquisition of antibiotic resistance resulting from natural mutations or transferring of resistant genes among each other (Ripanda et al. 2023; Ma et al. 2024; Ullah et al. 2024a, b; Khan et al. 2021). Incomplete metabolic processes, uncontrolled use of antibiotics and disposing medicines in water are the factors that contribute to the arising aquatic pollution (Kayode-Afolayan et al. 2022). Wastewater treatment facilities, additional to water purification plants, also help in dispersing the drug residuals in different environmental locations by releasing active ingredients (Mozhi et al. 2022; Foxman et al. 2024). Antibiotics are essential in treating human and animal bacterial infections as they either directly kill them or stop them from growing further.

Antimicrobial resistance (AMR) to commonly available antibiotics has increased significantly, making treatment for infectious diseases ineffective (Rahim et al. 2017; Mumtaz et al. 2024), and surgeries riskier (Munita and Arias 2016). AMR has been designated as a major threat by the World Health Organization (WHO). As per reports, it is assumed that by 2050 AMR will be responsible for 10 million deaths annually (Hill et al. 2013; Murray et al. 2022). Water systems contain multiple bacteria from the normal flora and provide shelter to resistant microbes that are the prominent hot spots for AMR development, proliferation, and spreading (Castañeda-Barba et al. 2024; Yang et al. 2023; Schmiege et al. 2024). Antibiotics are used in both home and hospital settings, when they are released into municipal waste they pave the path to develop resistance in microorganisms (Cantón et al. 2013). Recent study reports that consistent exposure to sub-inhibitory concentrations of antimicrobial (Noaman et al. 2023; Shaikh et al. 2023), and wastewater treatment plants maintain an optimum environment for antibiotic-resistant genes (ARGs) and antibiotic-resistant bacteria (ARB) to proliferate (Costa et al. 2023).

Furthermore, resistant microbes are prevalent in sewage water compared to un-polluted water, which is affirmative to human involvement to be substantially responsible for increased resistance in bacteria (Ram and Kumar 2020). Recent studies revealed that *E. coli* accounts for 86% of organisms producing resistant genes (Mustafai et al. 2023). The NDM (New Delhi metallo-beta-lactamase 1) gene was shown to be very prevalent in *Enterobacteriaceae*, followed by the CTX (A type of extended-spectrum beta-lactamase against Cefotaxime), KPC (*Klebsiella pneumoniae* Carbapenemase), and OXA-48 (Oxacillinase) genes. Similar results were found in another study carried out in Pakistan, where the NDM gene was shown to be the most common of all the genes that encode carbapenemase (CP) (Habib et al. 2022). It is alarming that CP and ESBL-producing (Extended-spectrum beta-lactamase) *Enterobacteriaceae* are becoming more common. Therefore, it is necessary to develop infection prevention techniques and perform broad national-level investigations to prevent the risk of a pandemic (Habib et al. 2022; Mustafai et al. 2023).

Recent studies undertaken by (Liu et al. 2023) and (Shao et al. 2024) underscores the growing significance of antibiotic resistance in wastewater by highlighting the potential and challenges of using wastewater surveillance to monitor antibiotic-resistant bacteria, emphasizing its importance for public health action. They provide a comprehensive review of small-scale wastewater-based epidemiology, demonstrating its effectiveness in tracking infectious diseases and antibiotic resistance. These recent studies illustrate the critical role of wastewater monitoring in understanding and mitigating the spread of antibiotic resistance, thereby enhancing our study’s relevance and contextual depth in the ongoing efforts to address this global health threat.

**Fig. 1:**
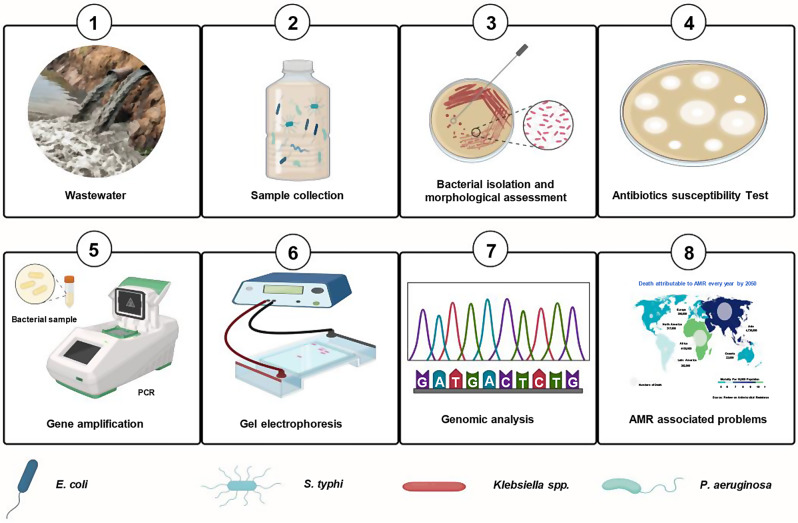
Schematic illustration of Characterization of Antibiotic Resistance Bacteria and Genes from Sewage Water. (1) wastewater (2) Sample collection (3) Bacterial isolation and morphological assessment (4) Antibiotic susceptibility test (5) Gene amplification (6) Gel electrophoresis (7) Genomic analysis (8) AMR associated problems

This research focuses on exploring the prevalence of multi-drug-resistant (MDR) bacteria within Bahawalpur city, nearby Sutlej River in Pakistan. The study aims at identifying specific resistance genes by employing both microbiological techniques and molecular analysis approaches. This includes the use of sequence alignment methods to trace and compare the genetic markers associated with resistance. This also study aims to add to the expanding body of knowledge on the environmental spread of antibiotic resistance by evaluating a wide range of bacterial isolates and defining their resistance profiles. This work is unique in that it examines both ARBs and ARGs in depth, offering a more complete picture of the region’s antibiotic resistance environment. Additionally, the research seeks to analyze the patterns of epidemiological distribution of antimicrobial resistance in the region, assessing how these resistant bacteria propagate and potentially impact public health (Fig. 1) (Fig. S3).

## Materials and methods

### Study area and significance

**Fig. 2:**
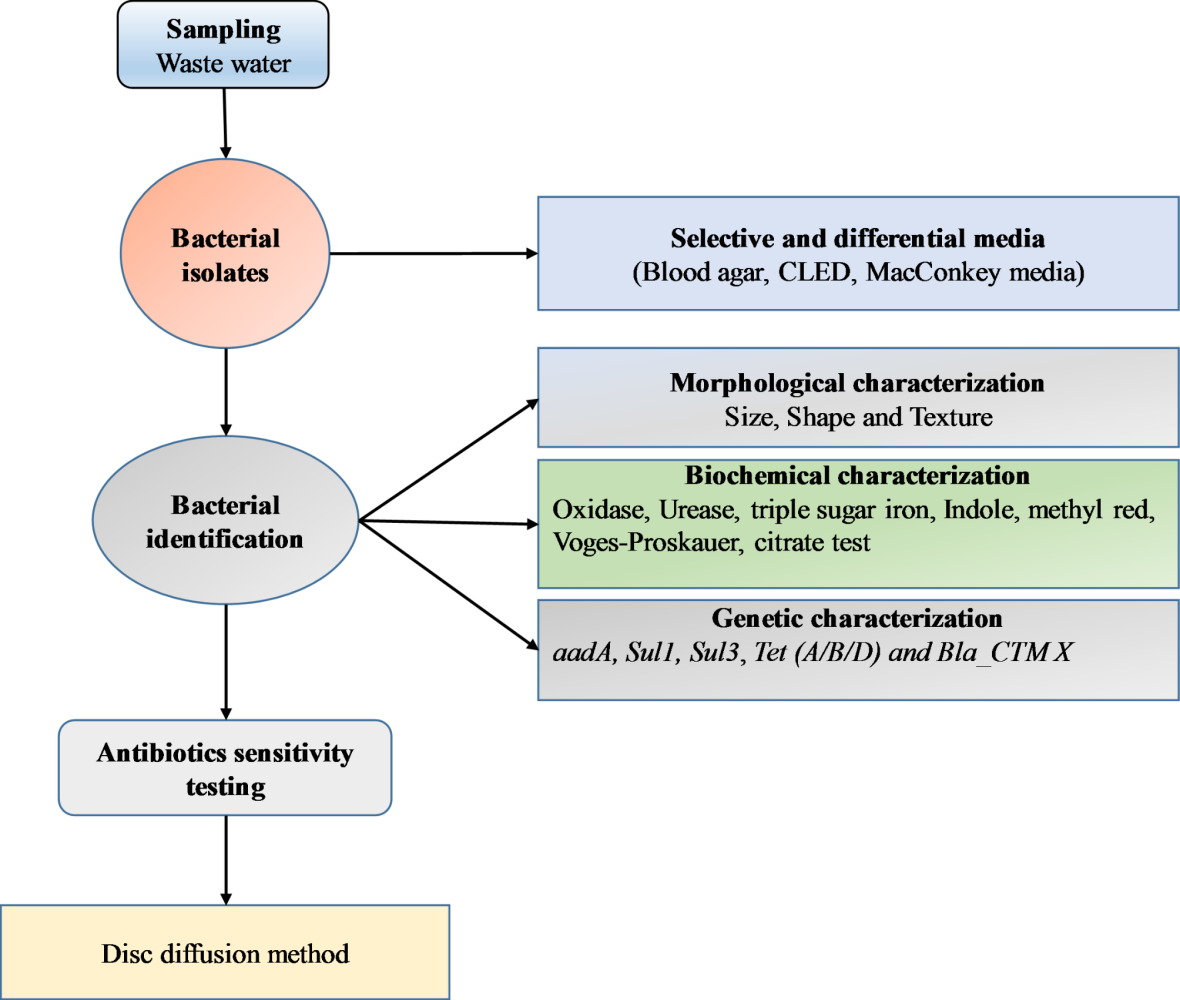
Schematic representation of Methodology. Biological samples from waste water were collected and cultured on growth media. Subsequently, identification approaches including morphological and biochemical identification tests were performed. Genetic characterization using a set of primers and sequence alignment was performed. Identified bacteria were subjected to culture sensitivity tests

The strategic selection of this particular region for intensive study is of great importance, offering a substantial opportunity to assess the sanitation standards of the city comprehensively. The sampling site is located at the junction of sewage water mixing with the fresh water reservoir, the Sutlej River. It is the point where the wastewater of the city meets with the river water. The sample was collected from both the inlets and outlets of the sewage water. Furthermore, it provides a significant diagnostic potential to uncover any environmental threats associated with the protracted transit of contaminants. Such pollutants are invariably a byproduct of the effluent discharge into the Sutlej River, a process that could carry implications for both local and downstream ecosystems. Thus, this is a promising study that looked deep into the hygienic profile of the city and in identifying possible risks linked to the long-haul movement of pollutants through the river’s course, as illustrated in Fig. 2.

### Sampling

#### Types of samples

In this cross-sectional study, a total of 36 water samples were collected from the Sutlej River at various critical points, including 26 from wastewater efflux and 10 from receiving waters. The study was conducted around the urban area of Bahawalpur, Pakistan, where the river collects effluents from treatment systems.

#### Sample collection and transportation

**Fig. 3:**
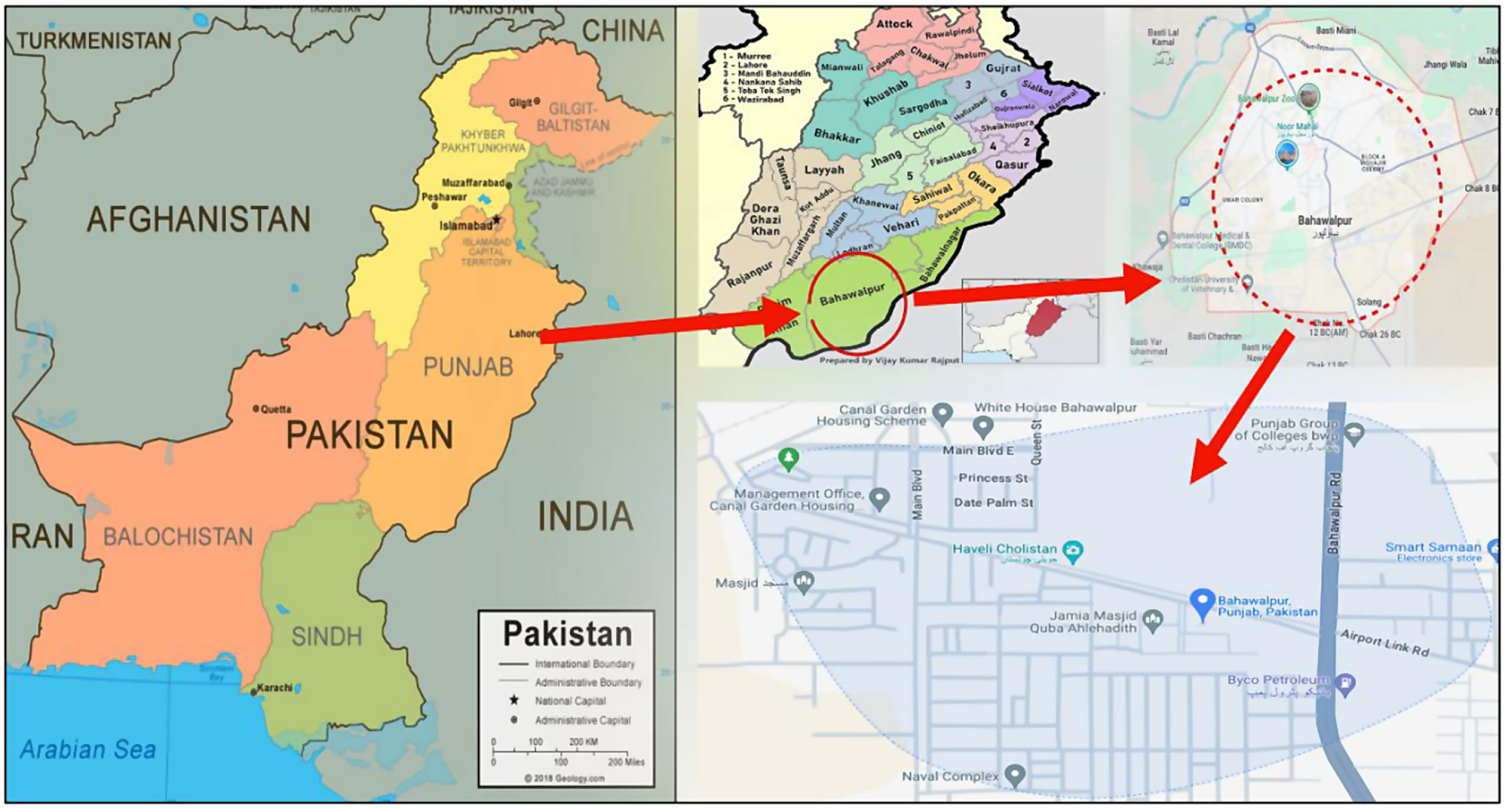
Geographic Mapping of Antibiotic-Resistant Bacteria in Sewage Waters of the Sutlej River, Bahawalpur District, Punjab Province, Pakistan. This map shows a detailed geographical representation, sampling locations where antibiotic-resistant bacteria were identified in the sewage waters of the Sutlej River

Sample collection involved the use of plastic containers that were sterilized with 70% ethanol and were pre-rinsed with the sampling water to avoid contamination and to adapt the containers to the water environment. Once collected, samples were immediately sealed in these sterilized containers and were protected from sunlight. They were transported in a cooler maintained at 4 °C to preserve their integrity and were analyzed within 24 h to ensure the reliability of the results. All the samples were processed according to the map as shown in Fig. 3.

### Microbiological analysis

#### Sample processing

The isolation procedure for bacterial strains consisted of several steps. Initially, 3 mL of the water samples were taken into tubes containing 5 mL of sterile peptone water, serving as a buffer. These tubes were subsequently incubated at 37 °C for 24 h to facilitate the enrichment of bacterial populations.

#### Cultural isolation and identification

Following this incubation period, a 1µL aliquot from each culture was streaked onto blood agar, MacConkey agar, and CLED agar plates, which were then incubated overnight at the same controlled temperature of 37 °C. This step preceded the process of sub-culturing to obtain pure bacterial colonies, which were thereafter subjected to an examination of their macro-morphological characteristics (Pintor-Cora et al. 2023; Ferguson et al. 2019).

#### Biochemical confirmation

Colonies that were suspected to be representative of *Salmonella spp*., *E. coli*,* K. pneumonia*, and *Pseudomonas spp.* were subjected to microscopic analysis and additional biochemical profiling. Including the oxidase test, urease test, triple sugar iron (TSI) test, and indole, methyl red, Voges-Proskauer, and citrate (IMViC) tests, were conducted by previously reported methods (Easrat Jahan and Aungshi 2023; Chauhan et al. 2020).

#### Antimicrobial susceptibility testing

After bacterial identification and isolation from each sample, the standardized Kirby-Bauer disk diffusion was applied to find the antimicrobial susceptibility of bacteria (Bashir et al. 2023). Inoculums were prepared by suspending the fresh culture of bacteria into 4-5mL of normal saline, and the turbidity was set to 0.5 McFarland standards. This suspension was spread over the surface of freshly prepared plates of Mueller Hinton agar with the help of a swab for having a confluent growth. The sensitivity testing was performed by placing the paper disks impregnated with a distinctive amount of antibiotics over the surface of the bacterial lawn. The susceptibility of the isolates was checked against 16 different antibiotics including Ciprofloxacin (CIP), Ampicillin (AMP), Imipenem (IMP), Gentamicin (CN), Co-Trimoxazole (COT), Tetracyclines (TETS), Ceftriaxone (CTR), Tazobactum/ piperacillin (TPZ), Doxycycline (DO), Amikacin (AK), Meropenem (MEM), Sulfamethoxazole (SXT), Ceftazidime (CAZ), Fosfomycin (FF), Cefhalexin (CEP) and Amoxicillin (AX). These plates were incubated aerobically at 37 °C for about 16–18 h according to the standard. Following the incubation period, inhibitory zone diameters were measured using a ruler, and the findings were categorized as resistant, sensitive, or intermediate sensitive according to the 2020 CLSI guidelines (Humphries et al. 2021). For every evaluated bacterial strain, quality control strains were included to guarantee the precision and reliability of the findings. The strains that were utilized as quality control were *Salmonella enterica* ATCC 14,028, *Pseudomonas aeruginosa* ATCC 27,853, *Klebsiella pneumoniae* ATCC 700,603, and *Escherichia coli* ATCC 25,922. The robustness of our results was guaranteed and the antibiotic susceptibility testing protocols were validated with the assistance of these quality control strains.

### Molecular analysis

#### DNA isolation

A heat-based method was used to isolate DNA from genome (Carriero et al. 2016). A 1.0 mL sample in Tryptic broth culture was first concentrated by centrifugation, then cleaned and resuspended in nuclease-free water. This suspension was then heated in a 95 °C water bath for 5 min before being rapidly cooled on ice for another 5 min. This heating and cooling cycle was performed twice, followed by centrifugation at 10,000 rpm for 10 min at 4 °C. This technique was selected because it preserves the integrity of the bacterial DNA while efficiently concentrating bacterial cells by settling them at the bottom of tube. Even tiny bacterial cells are effectively pelleted using centrifugation at 10,000 rpm, enabling a more precise and efficient DNA extraction process. The quality and concentration of the resulting DNA were evaluated using electrophoresis in a 1% agarose gel and DNA was quantified spectrophotometrically with a NanoDrop Lite Spectrophotometer, and was then stored at -20 °C for future use.

#### Molecular identification of bacterial species

Molecular analyses were performed on all identified samples, which were first detected using biochemical and phenotypic approaches (Elgendy et al. 2022). For PCR amplification, the universal primers were used that target conserved regions of the 16 S rRNA genes and intended to produce a product of around 1500 base pairs in size using gel electrophoresis.

#### Identification of resistant genes

PCR analysis was performed on the isolates exhibiting phenotypic resistance to determine various known resistance genes against different antibiotics. For these resistance genes, both positive and negative controls were used; however, it was not possible to get positive controls for all of the genes. In these situations, PCR techniques and previously reported primers were used (Ripanda et al. 2023; Abu-Sini et al. 2023). The reaction volume for PCR amplification was set at 25 µL, including 12.5 µL of 2X PCR Master Mix, 1 µL of each primer (10 µM), 1 µL of template DNA, and 9.5 µL of nuclease-free water. The cycling parameters are as follows: first denaturation was performed at 95 °C for 5 min, a total of 35 cycles of denaturation at 95 °C for 30 s, annealing at 55 °C for 30 s, and extension at 72 °C for 1 min were completed. Finally, a 10-minute extension at 72 °C was done. This study focused on the occurrence of the Sul1, Sul3, aadA, Tet (A/B/D) and Bla_CTM.X genes within the genomic DNA by using gene-specific primers as listed in Table S1. These genes were amplified using PCR and the products were run on 1.5% agarose gel.

#### Sequence alignment

Identifying antimicrobial resistance genes is critical for understanding the mechanisms and epidemiology of antibiotic resistance. In order to confirm phenotypic data and for epidemiological objectives, genotypic testing of isolates suspected of being resistant is frequently carried out (Zankari et al. 2012). PCR has been the most popular method for determining if certain genes are present. Frequently, just one or a small number of the genes involved in resistance are examined. This study used the Needleman-Wunsch alignment approach (Altschul 1997, 2012) to detect sequence similarities among the different resistance genes (Hendriksen et al. 2019). This investigation attempted to uncover the collective resistance pathways to commonly administered antibiotics.

The Needleman-Wunsch algorithm was implemented using the ClustalW software package. ClustalW is a popular multiple sequence alignment tool that utilizes a progressive approach to align multiple sequences simultaneously. The key steps involved in the sequence alignment process using ClustalW are as follows: The amino acid or nucleotide sequences of the resistance genes were provided as input to ClustalW. ClustalW initially calculates pairwise alignments between all pairs of sequences using the Needleman-Wunsch algorithm and a specified scoring matrix. A guide tree was constructed based on the pairwise alignment scores, representing the evolutionary relationships between the sequences. The sequences were progressively aligned based on the guide tree, starting with the most closely related pairs and gradually incorporating more sequences. The initial alignment was refined through iterative refinement steps to optimize the overall alignment score.

## Results

### Isolation and identification of antibiotic-resistant Bacteria

Morphological analysis of various bacterial species demonstrated distinct phenotypes. Notably, the identified colonies exhibited a greenish hue, medium to large size, and a central “cattle eye” appearance. Those colonies that failed to ferment lactose on MacConkey agar, gram staining revealed that these bacteria are gram-negative, short rods, typically arranged singly. Oxidase tests confirmed their positive reaction, identifying them as *P. aeruginosa* (Kollaran et al. 2019). Conversely, another group of bacteria on MacConkey agar formed pinkish, smooth, and shiny mucoid colonies of medium to large size. These did not cause hemolysis on blood agar, although some exhibited swarming behavior. These isolates were gram-negative, varied in cell size, and arranged singly. The bacteria suspected to be *P. aeruginosa* tested positive for oxidase, while those presumed to be *Salmonella spp*. negative (Ripanda et al. 2023). Additionally, isolates thought to be *E. coli* or *K. pneumoniae* showed positive results in IMViC and TSI tests (Nakkarach et al. 2020). The percentage of bacterial isolates from wastewater are shown in Table 1.

**Table 1:** Percentage of bacterial isolates from wastewater samples

Bacterial species	No. of isolates (*n* = 64)	Percentage (%)
*K. pneumoniae* *P. aeruginosa* *E. coli* *Salmonella spp.*	21 18 16 09	32.8 28.1 25.0 14.0

### Antibiotic susceptibility tests

Antibiotic resistance among the bacterial isolates was tested (Fig. S1). Notably, resistance was observed in 90% of *E. coli* isolates, 58% of *K. pneumoniae* isolates, 55% of *P. aeruginosa* isolates, and 53% of *Salmonella spp.* isolates against the six antibiotics assessed. All four bacterial species exhibited substantial resistance to ciprofloxacin. Conversely, resistance levels to imipenem and gentamicin were relatively lower, as outlined in the accompanying study data (Table 2).

**Table 2:** Antibiotic resistance of bacterial spp

Bacteria		Antibiotics				
	AMP (%)	CIP (%)	GN (%)	COT (%)	IMP (%)	TET (%)
*K. pneumoniae* (*n* = 21)	12 (57)	7 (33)	3 (14)	5 (24)	8 (38)	14 (67)
*P. aeruginosa* (*n* = 18)	0 (0)	10 (56)	12 (67)	18 (100)	0 (0)	0 (0)
*E. coli* (*n* = 16)	16 (100)	15 (93)	3 (19)	9 (56)	2 (13)	13 (72)
*Salmonella spp.* (*n* = 9)	5 (55)	7 (78)	0 (0)	6 (67)	1 (11)	0 (0)
**Total (%)**	**52**	**61**	**28**	**59**	**17**	**42**

AMP: Ampicillin, CIP: Ciprofloxacin, GN: Gentamicin, COT: Co-Trimoxazole, IMP: Imipenem, TET: Tetracycline

The results revealed significant variations in MDR rates among the species, with *E. coli* displaying the highest rate of resistance, followed by *K. pneumonia*, *P. aeruginosa*, and *Salmonella spp*. These findings underscore an alarming pattern of increasing antibiotic resistance within these bacterial groups.

### Molecular identification of resistance genes in bacterial species

Universal primers targeting the 16 S rRNA gene were employed to amplify DNA from a cohort of 17 bacterial isolates, facilitating their molecular identification. Genomic analysis revealed significant findings regarding antibiotic resistance genes among the bacterial samples. *K. pneumoniae* displayed the highest prevalence, accounting for 44% of all detected resistance genes, followed by *E. coli* and *Salmonella spp*., each contributing 24%. *P. aeruginosa* was found to harbor 8% of the resistance genes (Table 3).

**Table 3:** Occurrence of antibiotic-resistant genes detected on four bacterial species

Bacterial species	No	aadA	Tet (A/B/D)	Sul1	Sul3	Bla	MDR (%)
*K. pneumonia* *P. aeruginosa* *E. coli* *Salmonella spp.* Total (%)	8 3 4 2	3 2 1 1 41	1 0 2 2 29	2 1 2 1 35	3 0 1 2 35	2 0 0 0 12	44 08 24 24

A substantial proportion of the isolates, 70% (12 out of 17), were carriers of genes resistant to sulphonamides. Specifically, six isolates possessed the Sul1 gene, and another six carried the Sul3 gene. Additionally, 41% of the isolates (7 out of 17) tested positive for aminoglycoside resistance genes. Resistance to tetracyclines was observed in 29% of the isolates, with the presence of *Tet A/B/D* genes identified among them. β-lactamase genes, including *bla* CTX-M, were detected in 12% of the isolates (2 out of 17) (Fig. S2). Notably, *K. pneumoniae* was found to possess these genes more frequently than *E. coli*, as detailed in Table 3.

### Sequence alignments

**Fig. 4:**
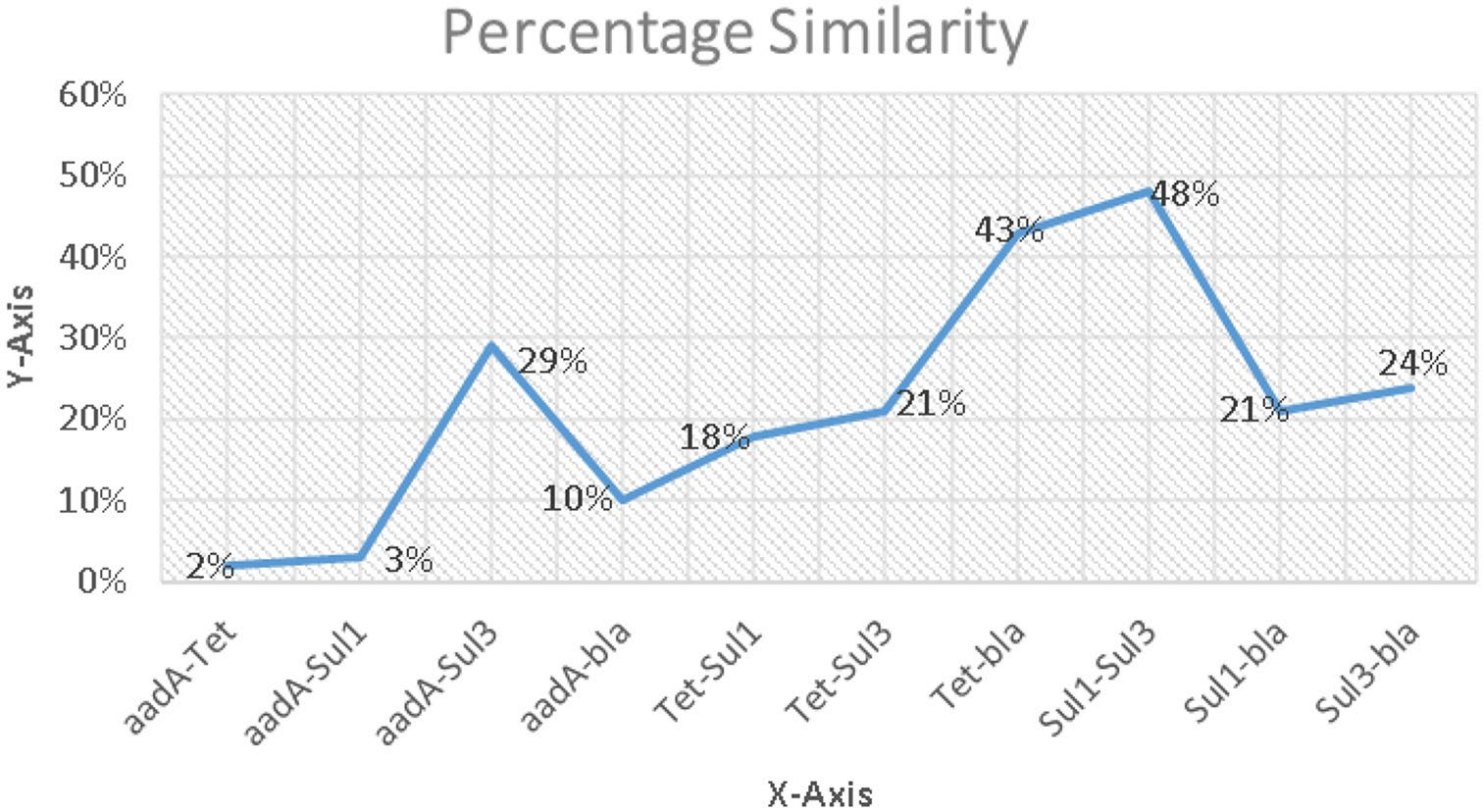
Similarity index of resistance-causing genes among each other. The highest similarity was observed between *Sul 1*-*Sul 3* followed by *Tet*-*bla*, *aadA*-*Sul 3*, *Sul 3*-*bla*, *Tet*-*Sul 3* and *Sul 1*-*bla* respectively

The sequence alignments results of resistant genes have shown different similarity patterns among multiple gene pairs being compared. The following gene pairs exhibited the highest degree of similarities, the resemblance scores of *Sul1-Sul3* and *Tet-bla* genes were 48% and 43% respectively. Following these pairings was *aadA-Sul3* gene pair that showed 29% conserved sequences. All the other gene pairs sequence alignments results displayed a few conserved regions placing the similarity degrees within a range of 2-24% (Fig. 4). These similarity patterns depict genetic resemblances and possible common resistance mechanisms among the genes being aligned. The diverse similarity patterns highlight the complexity of these genes, that emphasizes on the need of further studies in these areas to completely comprehend the resistance mechanisms that these genes confer against the commonly applied antibiotics. These mechanisms will also help in understanding the links and possible interactions among different resistant genes.

## Discussion

This research examined the presence of AMR bacteria in the Sutlej River, specifically targeting sections of the river that are impacted by urban wastewater discharge. The study aimed at determining how the influx of contaminants from municipal sources influences the proliferation and distribution of bacteria that are resistant to standard antibiotic treatments. This investigation is crucial for understanding the environmental consequences of urban waste on river ecosystems and assessing potential public health risks associated with waterborne AMR pathogens.

*P. aeruginosa* was identified on MacConkey agar as the non-lactose fermenting, gram-negative, short rods and tested positive for oxidase. In contrast, the pinkish, mucoid colonies on the same agar, exhibiting varied sizes and swarming behavior identified as gram-negative strains. Oxidase testing was pivotal in differentiating *P. aeruginosa* from the oxidase-negative *Salmonella spp*., which showed no hemolysis on blood agar. These findings are instrumental in the differential diagnosis of bacterial isolates using conventional identification markers (Ripanda et al. 2023). *P. aeruginosa* is a facultative bacterium, functioning as an aerobic pathogen affecting both humans and plants. The observed increase in AMRs among clinically significant bacteria could be linked to enhanced genetic exchange opportunities within the ambient antibiotic-resistance gene pool. Although many *E. coli* strains are harmless and beneficial to the human gut flora, but certain strains may cause symptoms such as abdominal pain, fever, diarrhea, and vomiting (Nakkarach et al. 2020). Similarly, *K. pneumoniae*, typically a non-pathogenic inhabitant of the human intestine and feces, can become pathogenic when it infects other body parts, potentially affecting the liver, lungs, brain, eyes, bladder, and blood (Guo et al. 2022).

The current analysis revealed that *K. pneumoniae* was the most prevalent bacterium, constituting 32.8% of the identified bacteria, followed by *P. aeruginosa* at 28.1%, *E. coli* at 25.0%, and *Salmonella spp*. at 14.0%. These organisms demonstrated resistance to at least three of the six antibiotics tested. Notably, ciprofloxacin encountered the highest resistance among all tested bacteria, whereas resistance to imipenem was comparatively lower. These findings are comprehensively detailed in Table 2. The results are in line with previous findings showing *K. pneumoniae* and *P. aeruginosa* showcasing the highest resistance followed by *E. coli* (Havenga et al. 2019; Abayneh et al. 2023).

The noticeable resistance to antibiotics, particularly to ciprofloxacin among *E. coli*, *K. pneumoniae*, *P. aeruginosa*, and *Salmonella spp*., is profoundly concerning. The fact that 90% of *E. coli* isolates and over half of the isolates from the other bacterial species demonstrate resistance to multiple antibiotics poses a significant challenge in managing infections caused by these pathogens. The lower resistance rates to imipenem and gentamicin, however, may indicate that these antibiotics retain efficacy against these bacterial strains. A comparative study highlighted that all 75 isolates expressing ESBL were multidrug-resistant, primarily against trimethoprim-sulfamethoxazole, ciprofloxacin, and gentamicin (Estaleva et al. 2021). This suggests a diminished effectiveness of these drugs in treating infections, presenting considerable challenges in clinical settings (Pan 2022; Pearl et al. 2022). Moreover, the widespread occurrence of MDR remains a severe public health issue.

The genomic analysis underscored the prevalence of antibiotic resistance genes within the bacterial isolates. Utilization of universal primers targeting the 16 S rRNA gene enabled the precise molecular identification of various bacterial species. Notably, *K. pneumoniae* emerged as the most significant carrier of antibiotic resistance genes, surpassing other species such as *E. coli* and *Salmonella spp*. The significant presence of sulphonamide, aminoglycoside, and tetracycline resistance genes is particularly alarming. Furthermore, the detection of β-lactamase genes, especially bla CTX-M, in a subset of the samples emphasizes the diverse resistance profiles exhibited by these bacteria. The proliferation of antibiotic-resistant genes is largely attributed to inadequate healthcare and sanitation practices, which inadvertently expose pathogens to antimicrobial agents. The study noted that both *E. coli* and *Salmonella spp*. are linked to two ampicillin resistance genes (*aadA*) and four tetracycline resistance genes (*tet A/B/D*), indicating a high prevalence of resistance genes among the evaluated bacteria. The distribution pattern of these genes varies between species, with *K. pneumoniae* exhibiting a higher frequency of β-lactamase genes compared to *E. coli* the results are in line with (Farhadi et al. 2021; Ghenea et al. 2022). This critical information enhances our understanding of the antibiotic resistance landscape and is essential for guiding future research and intervention strategies to address the growing issue of antibiotic resistance. The recent discoveries of antibiotic resistance genes suggest their potential spread among bacterial populations through various mechanisms, including bacteriophages and the uptake of free DNA from the environment (Klatte et al. 2017).

Antimicrobial resistance is further propagated in various environments as bacteria move through different media such as soil, air, water, and within human and animal populations (Li et al. 2022). Given the multitude of sources contributing to wastewater, including residential areas, pharmaceutical companies, and healthcare facilities, urban wastewater systems, encompassing both collection and treatment plants, are particularly vulnerable to disseminating antibiotic-resistant microorganisms (Fig. S3). Numerous studies have documented that wastewater harbors a diverse array of contaminants, including both illicit and prescribed drugs, pesticides, pharmaceuticals, and personal care products. These contaminants present significant challenges to effective wastewater treatment processes (Makaye et al. 2022).

The findings of this study have important public health consequences. The existence of ARBs and ARGs in wastewater implies that these pollutants may permeate natural water bodies and eventually reach drinking water sources. This might lead to a rise in the occurrence of difficult-to-treat illnesses due to a lack of antibiotic choices, creating a severe public health risk. Furthermore, the dissemination of ARGs by horizontal gene transfer might exacerbate the situation, potentially leading to the establishment of multidrug resistant bacterial species.

A variety of solutions can be used to reduce these risks. Initially, upgrading wastewater treatment techniques to properly remove ARB and ARGs is critical. This might entail modernizing current treatment plants with new technology including membrane bioreactors, enhanced oxidation processes, and bioaugmentation. Furthermore, stronger laws and monitoring of antibiotic usage in agriculture and hospital contexts are required to decrease the amount of antibiotics entering wastewater systems. Public awareness campaigns and stewardship initiatives can also help reduce inappropriate antibiotic use. Implementing these techniques together can assist to slow the emergence of antibiotic resistance and safeguard public health.

The relatively small sample size and geographical focus may limit the generalizability of our findings. A larger sample size would have allowed for a more comprehensive analysis of antibiotic resistance in the study area. Additionally, the study was limited to a specific region of the Sutlej River, and the prevalence of antibiotic resistance may vary in other regions. Future studies with larger sample sizes and broader geographical coverage could provide a more comprehensive understanding of antibiotic resistance in the region.

## Conclusions

This investigation highlights the crucial problem of antibiotic resistance in wastewater by identifying a range of ARB and ARGs in diverse wastewater sample types. This demonstrates the important threats to the environment and public health that wastewater poses as a reservoir and route for the spread of antibiotic resistance. Significant findings include the discovery of many clinically significant ARBs and ARGs, indicating that the efficacy of wastewater treatment procedures in removing these pollutants may be limited. The findings highlight the need for stronger antibiotic usage laws and better wastewater treatment technology in order to slow the spread of antibiotic resistance. The study also emphasizes how crucial it is to regularly monitor and detect ARB and ARG levels in wastewater in order to assess the success of applied measures and modify plans as necessary. This investigation came up with important outcomes that signify the collaborations among governmental, technical and public health activities. We can protect public health and water reservoirs by aligning our future research on streamlining treatment processes and finding innovative plans.

## Electronic supplementary material

Below is the link to the electronic supplementary material.

Supplementary Material 1

## Data Availability

The datasets used and/or analyzed during the current study are available in the manuscript.
