# Supplementary material for: Public health implications of antibiotic resistance in sewage water: an epidemiological perspective
Source: Bioresour Bioprocess. 2024 Sep 28;11(1):91. doi: 10.1186/s40643-024-00807-y (PMC11438758; doi:10.1186/s40643-024-00807-y)
Supplement: Supplementary file 1 — Supplementary Material 1 [file 40643_2024_807_MOESM1_ESM.docx]

**Supplementary Information**

**Public Health Implications of Antibiotic Resistance in Sewage Water: An Epidemiological Perspective**

**Kashif Rahim^1^****, Muhammad Naveed Nawaz^2^,** **Mazen Almehmadi^3^, Meshari A. Alsuwat^3^**, **Luo Liu^1^, Changyuan Yu^1^ and Shahin Shah Khan^1*^**

^1^ College of Life Science and Technology, Beijing University of Chemical Technology, Beijing 100029, China.

^2^Department of Biological Sciences and Technology, China University of Geosciences, Wuhan, China.

^3^Department of Clinical Laboratory Sciences, College of Applied Medical Sciences, Taif University, P.O. Box 11099, Taif 21944, Saudi Arabia.

***Correspondence:** shahinshah@buct.edu.cn

**Table S1**

Primers used to detect Aminoglycosides, Cephalosporins, Tetracyclines, Sulfonamides, and 16SrRNA.

| Antibiotics | Genes | Primers (5’-3’) | Temp | Size |
| --- | --- | --- | --- | --- |
| Aminoglycosides  Tetracyclines  Sulfonamides  Cephalosporins  Universal Primers | *aadA*  *Tet (A/B/D)*  *Sul1*  *Sul3*  *Bla_CTM.X*  *16s rRNA* | F-GGAGGTTGAGCAAAAAGCGG  R-CGCTTCCGTTTGGCATTGAA  F-TGCCTTGCAGTTCTCCCTG  R-CACCCACTACGGCATCCTG  F-CGATCGAAATGCTGCGAGTC  R-GCGGGGCTCAAGAAAAATCC  F-AGCATGCTCTGCATTTGGTTG  R-ACGCTTTACACCAGCCTCAA  F-AACTTTTACAGGAGGTGCATTG  R-TCATATCCTTTCGGTCCGCC  F-CACCCTCTGCAAAATCAAAAGGT  R-TCAAATTCGTAAAGGGCCTAATAAA | 60  59  59  60  57  59 | 703  893  457  413  426  518 |

**Figure S1.** Antibiotic resistance of bacterial strains using filter diffusion method.

**Figure S2.** PCR detection of AMR gene in isolates from waste water as visualized using agarose gel electrophoresis. *Bp* base pair, *M* molecular weight standard, (A) *Bla-CTMX* (426 Bp), (B) *16S RNA* (518 Bp), (C) *aadA* (703 Bp), (D) *Sul 1* (457 Bp) and (E) *Sul 3* (413 Bp).

**
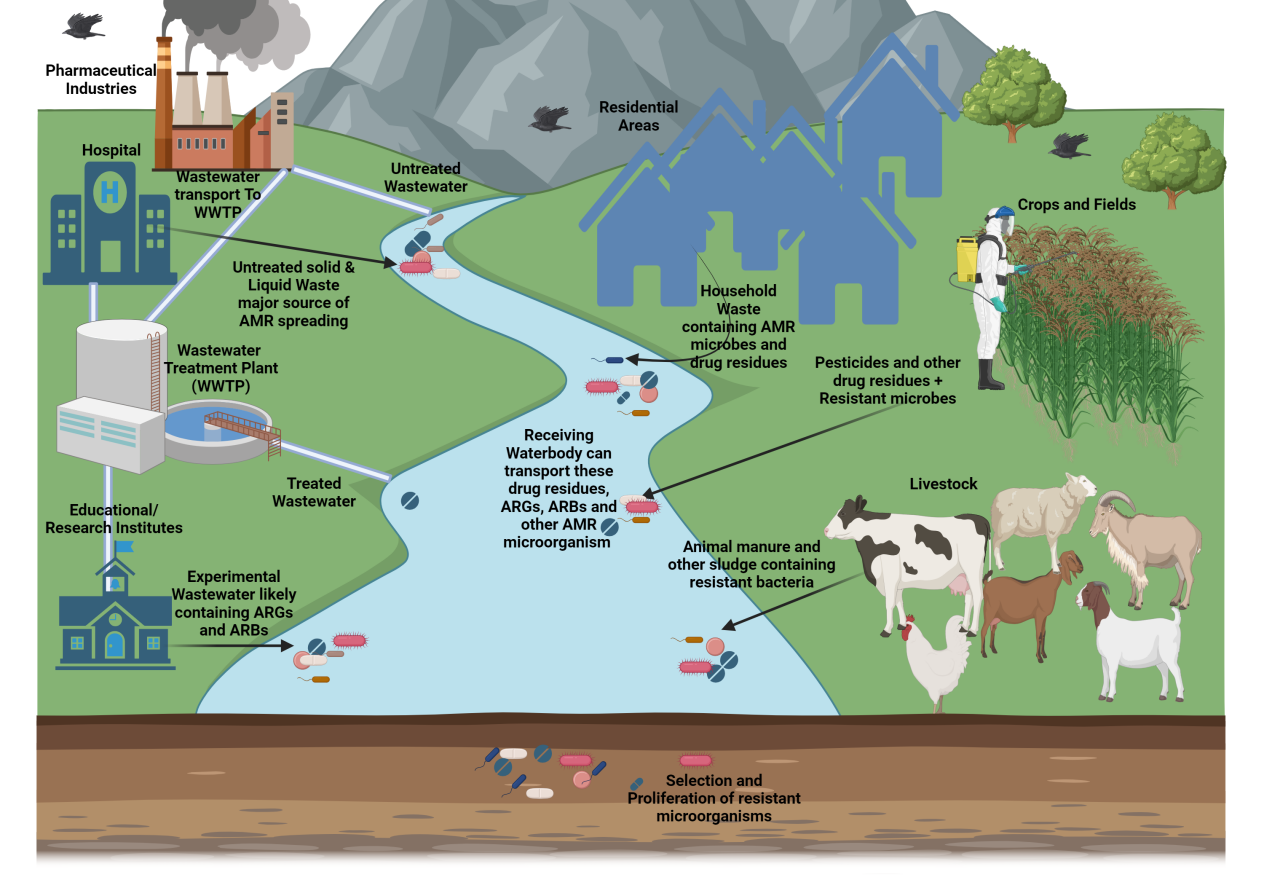
**

**Figure S3.** The image depicts how Antimicrobial Resistance (AMR) spreads, effluent from hospitals, pharmaceutical businesses, and residential areas, as well as experimental effluent from educational institutions, transfer AMR microorganisms, ARGs (antibiotic resistance genes), and ARBs (antibiotic-resistant bacteria) to bodies of water. Untreated solid and liquid waste from such sources has a key role in AMR propagation. Livestock and agriculture are vulnerable to AMR via animal waste and pesticide usage, which also spreads resistant bacteria.
